# Human pegivirus, Toscana virus and herpesviruses identified in cerebrospinal fluid from adults with unexplained neurologic disease, Spain, 2022–2023

**DOI:** 10.1099/jgv.0.002302

**Published:** 2026-07-17

**Authors:** Ana Donoso, Ana Belén Pérez, Marcos Lopez-Dosil, Ana Vázquez, Fabiana Gámbaro, María Paz Sánchez-Seco, Luis Martinez-Martinez, Maria Cabrerizo, David Tarragó, Maria Dolores Fernandez-Garcia

**Affiliations:** 1 Centro Nacional de Microbiología, Instituto de Salud Carlos III, Madrid, Spain; 2 Escuela de Doctorado, Universidad Autónoma de Madrid, Madrid, Spain; 3 Hospital Universitario Reina Sofia, Córdoba, Spain; 4 CIBER Infectious Diseases (CIBERINFEC), Madrid, Spain; 5 Instituto Maimónides de Investigación Biomédica de Córdoba (IMIBIC), Córdoba, Spain; 6 Hospital Universitario Puerta de Hierro, Majadahonda, Madrid, Spain; 7 CIBER Epidemiology and Public Health (CIBERESP), Madrid, Spain; 8 Spatial Epidemiology Lab (SpELL), Université Libre de Bruxelles, CP 264/03 Avenue F. Roosevelt 50, Brussels 1050, Belgium; 9 Universidad de Córdoba, Córdoba, Spain; 10 Hospital La Paz Institute for Health Research (IdiPAZ Foundation), Madrid, Spain

**Keywords:** central nervous system infections, herpesviruses, human pegivirus, Toscana virus, viral metagenomics

## Abstract

Viral central nervous system (CNS) infections in adults frequently remain unresolved after routine diagnostic testing. We applied probe-based viral metagenomic next-generation sequencing (vmNGS) to cerebrospinal fluid samples from adults with suspected CNS infection and negative conventional diagnostics in a retrospective multicentre study conducted in Spain between 2022 and 2023. Among 40 idiopathic cases, vmNGS detected viral sequences in 6 patients without evidence of coinfection: human pegivirus (HPgV, *n*=3), Toscana virus (TOSV, *n*=1), herpes simplex virus type 1 (HSV-1, *n*=1) and varicella-zoster virus (VZV, *n*=1). Two HPgV-positive patients were transplant recipients, with neurological disease occurring more than 2 years after transplantation, compatible with possible long-term viral persistence in immunocompromised hosts. TOSV genotype B was identified in a patient residing in central Spain, supporting consideration of TOSV in selected cases of unexplained aseptic meningitis during the vector season, including outside traditionally recognized Mediterranean coastal regions. Furthermore, the failure of syndromic panel testing to detect HSV-1 and VZV highlights the need for complementary diagnostic strategies when clinical suspicion remains high. Overall, the detection of unexpected viral sequences, together with missed clinically actionable infections, supports the use of complementary molecular testing in selected cases of unexplained CNS syndromes when routine diagnostics are negative. These findings highlight the added diagnostic value of vmNGS and provide sequence-level data for future studies of viral diversity and molecular epidemiology in neurological disease.

## Data Availability

Genome sequences have been deposited in the NCBI database under accession numbers PX663398 (TOSV segmL ADA155), PX663399 (TOSV segmM ADA155), PX682246 (TOSV segm S ADA155), PX663397 (HPgV ADA18), PX663400 (HPgV ADA63), PX663401 (HPgV ADA46 5′UTR), PX663402 (HPgV ADA46 E1/E2) and PX712187 (HSV1 ADA41).

## Introduction

Viral infections of the central nervous system (CNS) represent a significant cause of morbidity and mortality worldwide, often presenting as meningitis, encephalitis or myelitis [1]. While some cases are mild, many carry a high risk of neurological sequelae, especially if diagnosis and treatment are delayed. In a significant proportion of cases (ranging from 43% to 81%), the causative agent remains unidentified using conventional diagnostic methods such as PCR and serology [2,3].

Diagnosing viral CNS infections involves several challenges. One major challenge is the wide range of possible causative viruses, which cannot be identified based on clinical symptoms alone, requiring specific tests that may be costly or unavailable. Additionally, molecular or antigen-based tests might fail if the pathogen has undergone genetic changes or is an unexpected or emerging virus. Timing also matters, as the viral load in cerebrospinal fluid (CSF) can fluctuate, affecting the likelihood of detection depending on when the sample is taken [4,5].

The emergence of viral metagenomic next-generation sequencing (vmNGS) has provided a powerful tool to overcome these limitations, enabling the identification of known and novel viruses directly from clinical samples. Globally, studies have demonstrated the added value of this technique in identifying previously undetected viruses in neurologic cases with negative results using clinical routine diagnostic methods [4,7]. However, challenges remain, such as distinguishing true pathogens from contaminants and interpreting results in the context of low viral loads or partially degraded viral genomes [5].

In Spain, few studies have investigated viral CNS infections of unknown origin using vmNGS, and most have focused on paediatric populations [4,8, 9]. To address this gap, we conducted a retrospective study applying vmNGS to CSF samples from adults with CNS infections of unknown aetiology after comprehensive routine diagnostic testing. The study was designed as a descriptive genomic investigation aimed at generating sequence-level evidence of viral genomes present in CSF, characterizing viral genetic diversity where possible and assessing the added diagnostic value of vmNGS for unresolved CNS infections.

## Methods

### Study design and sample collection

A multicentre hospital-based retrospective study was conducted in two public tertiary care hospitals: Hospital Universitario Puerta de Hierro (HUPH) in the Madrid region, central Spain, and Hospital Universitario Reina Sofia (HURS) in the Andalusian region, southern Spain. CSF samples were collected during 2022–2023 from adult patients with suspected viral CNS infections in whom extensive routine diagnostic testing yielded negative results (idiopathic samples). Inclusion was based on the availability of residual CSF volume after completion of standard diagnostic procedures.

### Routine diagnostic methods

Laboratory workup slightly differed between the two hospitals. Both centres evaluate CSF samples from patients with suspected CNS infection using routine bacterial and fungal cultures and the FilmArray^®^ Meningitis/Encephalitis Panel, a multiplex real-time PCR detecting the most common bacteria, fungi and viruses involved in meningitis and encephalitis [bacteria (*Streptococcus pneumoniae*, *Neisseria meningitidis*, *Haemophilus influenzae*, *Listeria monocytogenes*, *Streptococcus agalactiae* (Group B) and *Escherichia coli K1*), fungi (*Cryptococcus neoformans*/*Cryptococcus gattii*) and viruses (herpes simplex virus 1 (HSV-1), herpes simplex virus 2 (HSV-2), varicella-zoster virus (VZV), enterovirus (EV), human parechovirus, cytomegalovirus (CMV) and Epstein-Barr virus (EBV))]. At HURS, testing for suspected West Nile virus or Toscana virus (TOSV) is sent to the Regional Reference Center (Hospital Virgen de las Nieves, Granada, Spain). Negative CSF samples with sufficient volume for vmNGS were stored at −20 °C and, when pooled, referred to the CNM. At HUPH, the FilmArray^®^ ME Panel is performed selectively based on clinical suspicion and disease severity. Negative CSF samples at HUPH are normally referred to the CNM for molecular testing of specific viruses (EBV, CMV, HSV-1/2, VZV, EV and HHV-6/7/8) depending on clinical suspicion [8,10]. Residual samples are stored in the CNM biobank at −20 °C. Those with sufficient volume were subsequently selected for vmNGS analysis.

### vmNGS library preparation and sequencing

The study samples comprised the following: (i) analytical positive controls consisting of archived CSF and stool specimens with previously confirmed viral detection by reference molecular methods and sufficient residual volume. These positive controls were selected from the CNM collection to represent both RNA and DNA viruses and to assess the overall performance of the vmNGS workflow, including nucleic acid extraction, library preparation, hybrid-capture enrichment, sequencing and bioinformatic detection. They were used exclusively as workflow controls; therefore, the positive-control panel was not intended to include all viruses potentially detectable by this broad agnostic approach. RNA virus controls comprised TOSV (*n*=1), EV (*n*=2) and norovirus (*n*=1), whereas DNA virus controls included human herpesvirus 6 (HHV-6, *n*=2), human herpesvirus 7 (HHV-7, *n*=1) and adenovirus 41 (*n*=1); (ii) negative controls included water samples and CSF samples from patients with no clinical suspicion of infection, as determined by the attending physician (stroke, epilepsy, depression, amyotrophic lateral sclerosis and multiple sclerosis), hereafter referred to as ‘negative CSFs’ (*n*=10); (iii) idiopathic samples from patients with suspected CNS infection of unknown aetiology after routine laboratory testing (*n*=40). Idiopathic samples were from HUPH (*n*=18) and HURS (*n*=22). Sample processing involved total RNA extraction from 200 µl of CSF using the QIAamp MinElute Virus Spin Kit (Qiagen). RNA was quantified by QuantiFluor RNA System (Promega), and fluorometric measurement was performed for normalization. vmNGS libraries were prepared using the NEBNext^®^ Ultra^™^ II Directional RNA Library Prep Kit for Illumina (New England BioLabs Inc., USA). Samples were dual-indexed using NEBNext^®^ Multiplex Oligos for Illumina (New England BioLabs Inc., USA). The initial volume for the vmNGS libraries was 5 µl at 1 ng µl^−1^, and 10 µl was used for CSF samples with very low input amounts following the manufacturer’s recommendations. Target enrichment was performed by hybrid capture using the Twist Comprehensive Viral Research Panel v2 (Twist Biosciences, San Francisco, USA) that covers a wide range of viruses (>3,000 known species). Twist post-capture library pools were PCR-amplified for 16 cycles, then quantified by QuantiFluor ONE dsDNA System (Promega, Madison, USA) on the Quantus Fluorometer (Promega) and finally quality-verified for sequencing with the Bioanalyzer High Sensitivity DNA Analysis System (Agilent Technologies, Inc., Santa Clara, USA). Enriched libraries were subjected to 2 NGS runs with the Illumina technology on NovaSeq-6000 and Nextseq-500 instruments (Illumina Inc., San Diego, CA, USA).

### vmNGS sequencing data analysis

Illumina sequencing reads were analysed as previously described [11]. Briefly, FASTQ files were analysed with the viralrecon pipeline (https://github.com/nf-core/viralrecon). Raw data were initially pre-processed using FastQC v0.11.9 and fastp v0.20.1 for quality control and adapter removal, filtering of short reads and trimming of low-quality bases. The resulting reads were analysed with Kraken2 v2.1.2 to remove human sequences. Non-host reads were assembled *de novo* with SPAdes (metaSPAdes mode). Afterwards, contigs were taxonomically annotated by blast against the NCBI viral database. Bowtie2 was used to map the filtered and clean reads after the pre-processing stage to map them against viral reference genomes. From the alignment step, we obtained the coverage depth and statistical metrics using SAMtools v1.19. FASTQ files were also processed on the IDseq platform (https://czid.org/), a cloud-based open-source platform for pathogen detection from metagenomic data [12]. A background model generated from negative CSF and water samples was applied to remove taxa commonly associated with environmental, reagent-derived or commensal viral contamination. A virus ‘hit’ was defined as a viral taxon with the highest number of reads aligning to both the nucleotide (NT) and non-redundant protein (NR) databases after background subtraction. To distinguish true hits from such background noise, viral reads were normalized to reads per million (r.p.m.). Additionally, we used the z-score as a statistical measure to assess the significance of viral presence in the sample relative to the background. Therefore, for optimal prediction of true positive result taxa with z-score <1, no assembled contigs (or contigs <500 bp) and <500 r.p.m. were excluded from further analysis [7,11, 13]. To increase confidence in viral detection, we additionally required >10% genome coverage distributed across multiple non-contiguous regions of the viral genome [13,14]. After applying all filtering criteria, the resulting viral hits were visualized in a heatmap constructed in RStudio [15] using normalized r.p.m. values.

### Phylogenetic analysis

To assess the phylogenetic relationships of the genomes detected in this study, sequence analyses were conducted. For this aim, we selected similar sequences available in GenBank and identified by using blast. Multiple sequence alignment was carried out using ClustalW multiple alignment program within the BioEdit Sequence Alignment Editor package, version 7.0.9.0, and the Multiple Alignment using Fast Fourier Transform v7.467. Maximum likelihood (ML) phylogenetic trees were reconstructed with Molecular Evolutionary Genetics Analysis (mega11) software package using a General Time Reversible (GTR) model and IQ-TREE 2.2.0 [16] using default settings, and the best-fitting substitution model identified by ModelFinder [17]. In both cases, this was followed by ultrafast bootstrap approximation [18] with 1,000 replicates.

### Confirmatory testing of vmNGS hits

TOSV was assessed by using conventional reverse transcription polymerase chain reaction (RT-PCR) as previously described [19]. Detection of human pegivirus (HPgV) and VZV was performed using in-house RT-PCRs (Table 1). HSV-1 was assessed by using quantitative real-time PCR (qPCR) as previously described [8].

**Table 1.** Nucleotide sequences of primers designed for HPgV and VZV

Primer	Nucleotide sequence (5′–3′)	Region*	Amplicon (bp)	PCR cycling conditions
HPgV-Fw1	GCT GTG CCC TTC GTC AAY AGG	1668–1688	735	50 °C 30 min; 95 °C 15 min; 35× (95 °C 1 min, 55 °C 1 min, 72 °C 1 min); 72 °C 10 min
HPgV-Rev1	CYC CCC GAG CGA GCT TCC	2386–2403		
HPgV-nFw2	GGT CCT ACA CCA TGA CCA AGA T	1765–1786	322	95 °C 15 min; 35× (95 °C 1 min, 52 °C 1 min, 72 °C 1 min); 72 °C 10 min
HPgV-nRev2	GTT GCC HGC ATC CAC CTC C	2069–2087		
VZV-OKAF	GGA CGT ACA CGT GAT ACT GAG AC	106223–106245	116	98 °C 30 s; 35× (98 °C 10 s, 60 °C 15 s, 68 °C 30 s); 72 °C 5 min
VZV-OKAR	AGT ATC TAG GCT CGC GGT TG	106320–106339		

*Nucleotide positions in reference to the sequence numbering of HPgV genotype 2 (GenBank accession number U45966) or VZV, strain Dumas (GenBank: X04370.1).

## Results

### CSF samples for vmNGS analysis and patient characteristics

The study included a total of 58 CSF samples, consisting of positive controls, negative controls and idiopathic cases. Clinical and demographic characteristics of patients with idiopathic CSF samples are summarized in Table S1 (available in the online Supplementary Material). Most samples were from adults with suspected meningitis (10/40; 25%), encephalitis (9/40; 22.5%), meningoencephalitis (6/40; 15%) or myelitis (6/40; 15%). The remaining cases comprised other neurological presentations, including encephalopathy, inflammatory CNS lesions, peripheral nerve involvement or seizure-related presentations, as detailed in Table S1. The median age was 60 years [interquartile range (IQR): 46.8–72.8 years], and 70% of patients were male.

### Viral identification by vmNGS and confirmatory PCR analyses

**Fig. 1.**
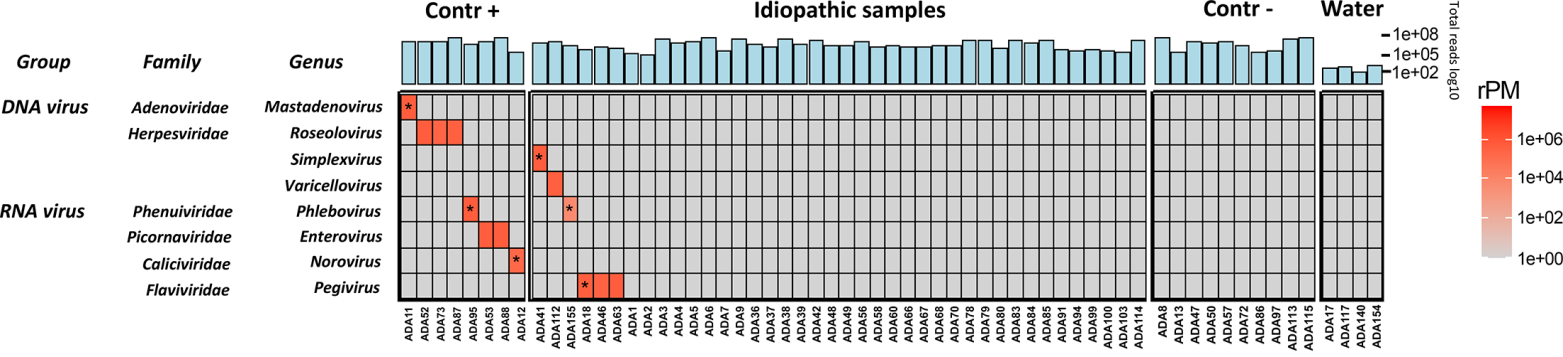
Heatmap of taxa identified in CSF from patients with CNS infections of unknown aetiology by metagenomic sequencing after negative results in routine clinical diagnostics, Andalusia and Madrid region, Spain, 2022–2023. The heatmap was set to ≥500 r.p.m., >1 contig, minimum genome size ≥500 bp, >10% genome coverage and z-score ≥1 above background to assure that the taxon does not appear in any of the samples in the background model. Each column represents idiopathic samples from patients with CNS infections, samples testing positive for specific viral agents (positive controls), negative samples from patients with non-infectious diagnoses and water samples. Each row corresponds to a viral genus detected in at least one sample. Heatmap was generated based on r.p.m., and the coloured scale represents r.p.m., with darker red indicating higher r.p.m. values. Accordingly, each red box indicates the number of r.p.m. for that viral genus in the sample, while grey boxes indicate samples in which no virus was detected. The asterisk denotes viruses with near-full-length genomes identified in samples by vmNGS. The base 10 logarithm of the total reads for each sample is shown on top. Coloured logarithmic scale represents r.p.m., with darker red representing the highest r.p.m.

**Fig. 2.**
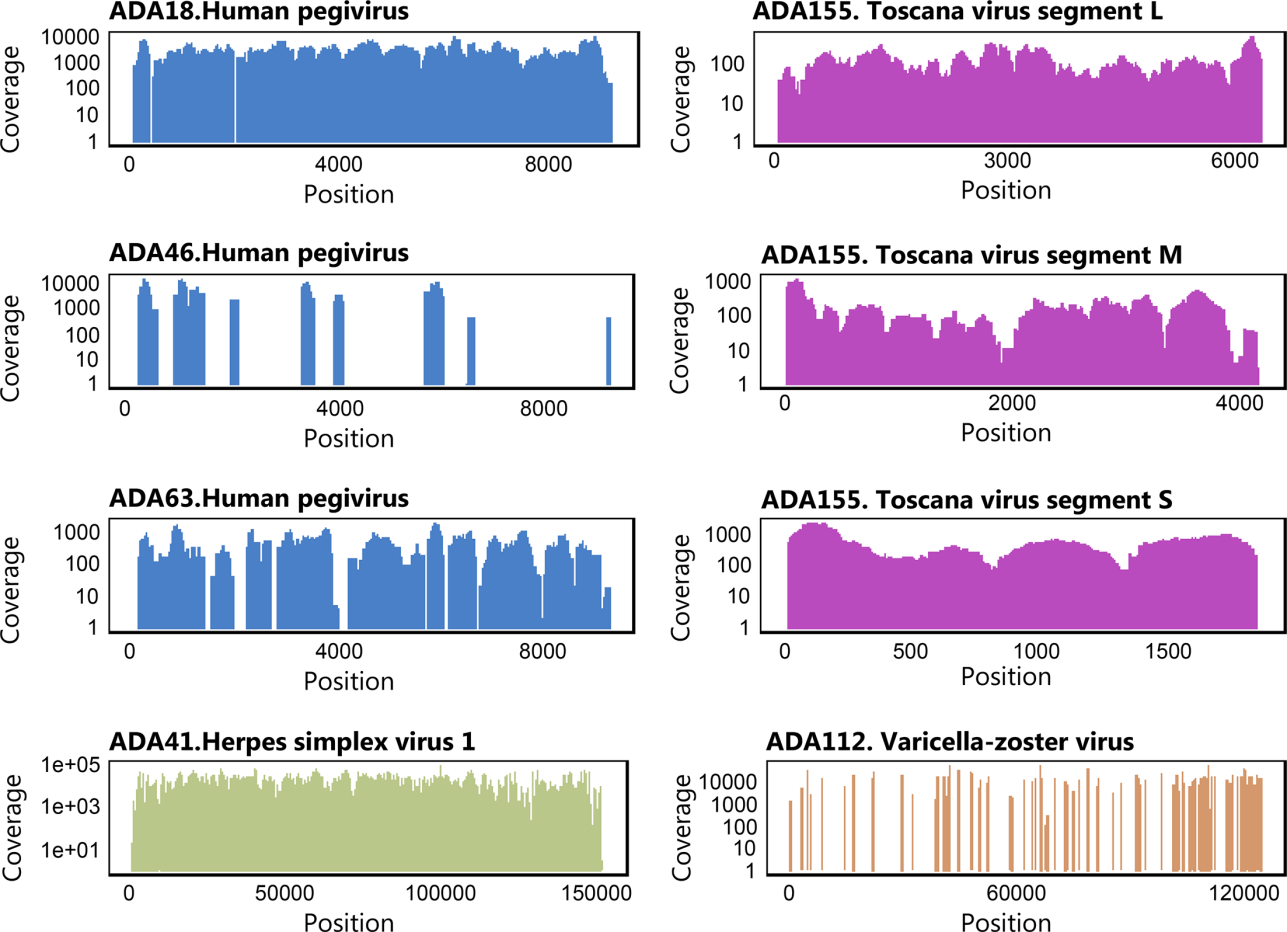
Sequencing depth coverage plots of near-full-length and partial genomes from viral hits obtained through vmNGS with probe-based enrichment. The Y-axis represents the log base 10 of the number of reads mapped to each position along the reference sequences (depth of coverage). The X-axis shows nucleotide positions.

Samples underwent nucleic acid extraction, library preparation, target enrichment by capture and sequencing. Excluding controls, a total of 486 million paired-end reads were obtained. After pre-processing, a mean of 12.2 million reads/sample was obtained (IQR 1–20.3 million). To summarize viral hits, a heatmap was generated showing the presence and relative abundance of viral reads, expressed as r.p.m. (Fig. 1). In positive-control samples, vmNGS correctly identified the previously diagnosed RNA and DNA viruses, including TOSV, EV, norovirus, HHV-6, HHV-7 and adenovirus 41. In negative-control samples, no potential viruses were found in the sequencing data (Fig. 1). Among the 40 idiopathic samples, 6 met the established criteria for a viral hit, with viral sequences identified for HSV-1 (*n*=1), TOSV (*n*=1), VZV (*n*=1) and HPgV (*n*=3). No other viruses were detected in these samples. Results were consistent across independent extractions and sequencing runs, supporting the robustness of the findings. Metagenomic assembly enabled the reconstruction of several viral genomes with high coverage and depth (Table 2). Nearly complete genomes were obtained for TOSV (segments L, M and S, 98–100% coverage), HSV-1 (93.7% coverage) and two HPgV genomes (ADA18 and ADA63, with 97.6 and 87.8% coverage, respectively), whereas HPgV ADA46 yielded only short fragments distributed across the genome (23.2% coverage; median depth 3,742×). The VZV genome was partially recovered (12.1% coverage; median depth 8,736×). In all samples, including those with partial genomes, the reads mapped across the genome and coverage depth values support a true positive signal [14] (Fig. 2). To further validate the vmNGS findings, we confirmed the presence of HPgV, TOSV and VZV in the original samples by Sanger sequencing of conventional RT-PCR products, while HSV-1 was confirmed by qPCR (Ct=23.74) (Fig. S1). The HSV-1-positive sample was obtained from a patient with encephalitis and brain MRI findings suggestive of possible herpetic encephalitis, whereas the VZV-positive sample was obtained from a patient with myelitis and cutaneous herpes zoster lesions suggestive of VZV-associated disease. Table 2 summarizes the characteristics of virus-infected patients.

**Table 2.** Characteristics, clinical details, routine laboratory results and metagenomic findings from CSF samples of patients with CNS infections of unknown aetiology, Spain, 2022–2023 (*n*=6)

Hospital	Sample ID	Age group	Sex	Month/year	Clinical suspicion	Clinical information	Routine clinical laboratory tests with negative results	CSF analysis			Genome length (nt)/cov. %/median cov. depth	r.p.m.	Virus hit
								WBC mm^3^	Lymph %	Prot mg/dl			
HURS	ADA41	60–69	M	Mar. 2023	Encephalitis	Brain MRI: suggestive of possible herpetic encephalitis; pneumococcal pneumonia and septic shock	BCU; FCU; FA-ME	372	69	73.8	152151/97.6/4858	999001	HSV-1
HURS	ADA46	50–59	M	Apr. 2023	ME	Patient with severe aplastic anaemia associated with systemic lupus erythematosus. Haematopoietic stem cell transplant (2021). Confusion and epileptic seizures; normal cranial CT and brain MRI	BCU; FCU; FA-ME; cryptococcal antigen	80	89	93.2	2181/23.2/3742	590142	Pegivirus
HURS	ADA63	20–29	F	Jan. 23	Meningitis	Admitted after 5 days of headache, fever and vomiting. Favourable evolution. No relevant medical history; not immunocompromised.	BCU; FCU; FA-ME	252	92	56.3	8247/87.8/314	162923	Pegivirus
HURS	ADA112	40–49	M	Nov. 2023	Myelitis	Central and peripheral demyelination on rituximab, presenting with acute lower limb numbness. Cutaneous herpes zoster lesions suggested VZV-associated myelitis. Normal brain MRI	BCU; FCU; FA-ME	12	100	157.9	125459/12.1/8736	718132	VZV
HUPH	ADA18	60–69	M	Mar. 2023	Multiple neuritis	Liver transplant (2020). Received >50 red blood cell transfusions, the most recent in Dec 2020. Admitted for assessment of multiple neuritis involving cranial nerves.	BCU; FA-ME	70	99	59.5	9136/97.6/2554	809252	Pegivirus
HUPH	ADA155	60–69	F	Jun. 2022	Meningitis	Malaise and headache with a 4–5-day duration. No recent travel or insect bites were reported	BCU; FA-ME; EBV, CMV, HSV, VZV, EV, HHV-6-7-8 PCRs	109	50	91.1	Seg-L: 6360/100/96 Seg-M: 4203/98.5/124 Seg-S: 1871/98.2/467	8605	TOSV

HUPH in the Madrid region; HURS in Andalusia.

BCU, CSF bacterial culture; CMV, cytomegalovirus; Cov., coverage; CSF, cerebrospinal fluid; CT, computed tomography; EBV, epstein-barr virus; EV, enterovirus; F, female; FA-ME, FilmArray® Meningitis/Encephalitis Panel; FCU, CSF fungal culture; HHV, human herpesvirus; HSV-1, herpes simplex virus 1; M, male; ME, meningoencephalitis; MRI, magnetic resonance imaging; nt, nucleotide; r.p.m., number of reads that align to a specific viral genome per million reads; VZV, varicella-zoster virus; WBC, white blood cells.

### Characterization of HPgV-positive samples

**Fig. 3.**
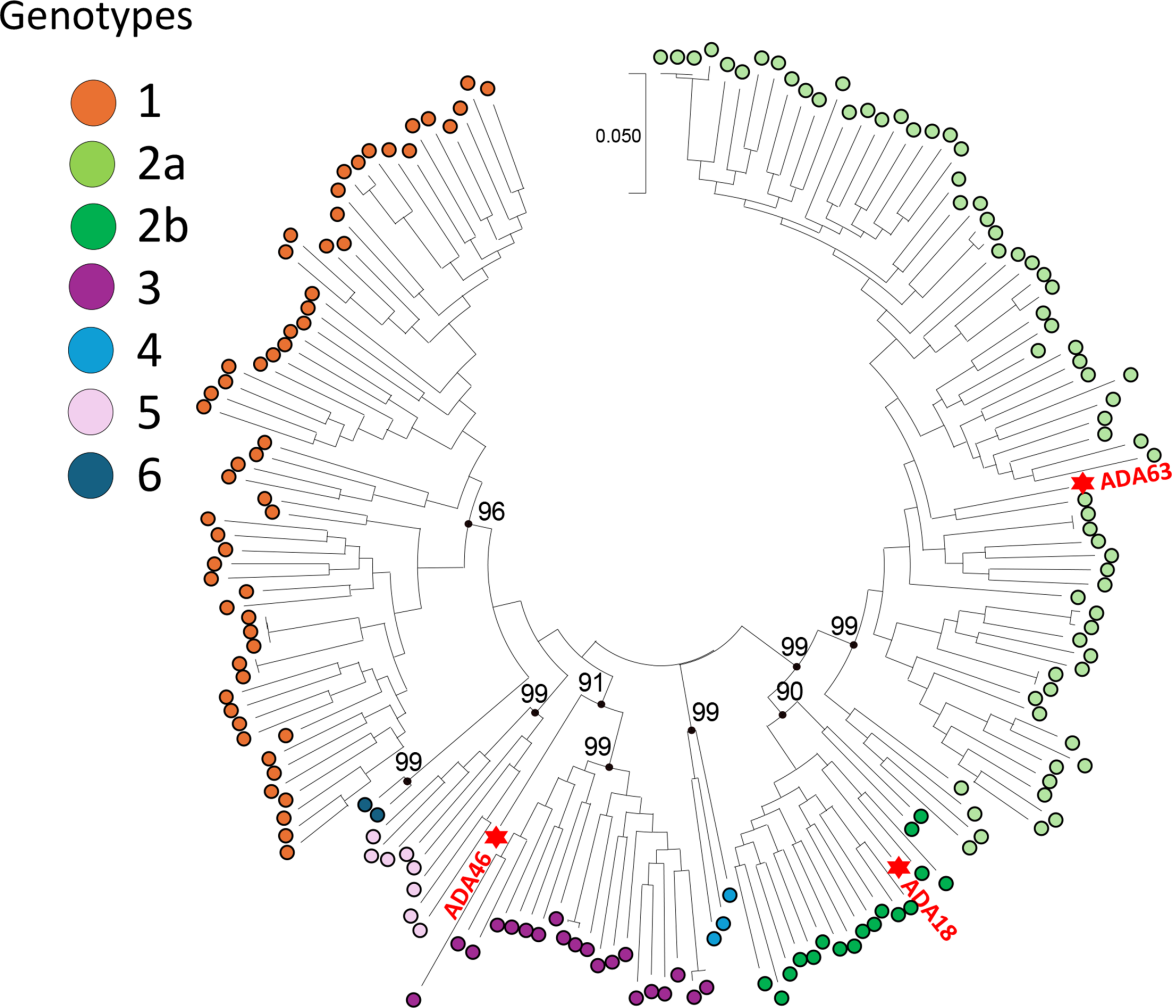
ML phylogenetic analyses of HPgV sequences. Phylogenetic analyses were conducted using complete GenBank reference sequences representing HPgV genotypes 1–6 to assign genotypes. Near-full-length sequences from ADA18 and ADA63 and a partial sequence from ADA46 were compared with reference sequences. The genotype of reference sequences is shown in the legend. Node support was estimated by 1,000 bootstrap replicates, and evolutionary relationships were inferred using the GTR model. Only bootstrap values above 80% are shown.

The genotypes of the detected HPgVs were determined by generating ML phylogenies of the genomes obtained in this study, together with a set of publicly available sequences representing the six known genotypes. The analyses placed the ADA18 sequence within genotype 2b, while ADA63 clustered into genotype 2a, both with high support (Fig. 3). For sample ADA46, although genome recovery was limited (23% coverage), the partial sequence obtained clustered within genotype 3, showing the highest similarity to genotype 3 strains from China and Japan (Fig. S2).

### Characterization of TOSV-positive sample and phylogenetic analysis

**Fig. 4.**
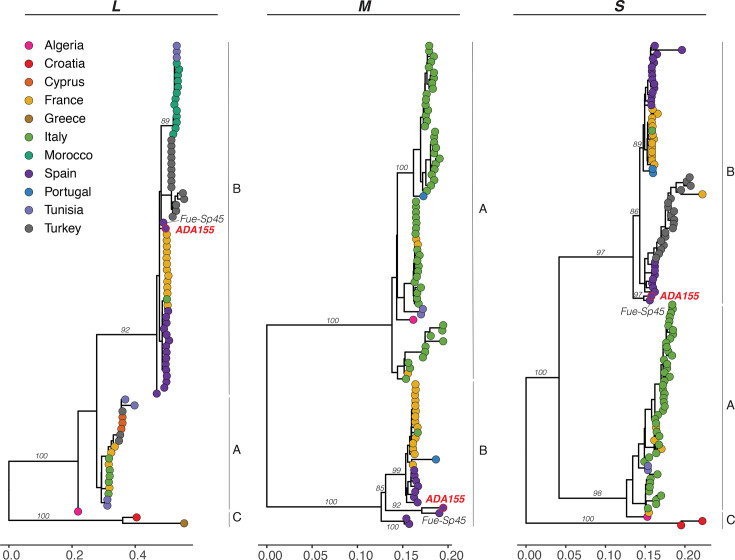
ML phylogenetic trees of TOSV segment L, segment M and segment S sequences. The different lineages are indicated. The sequence generated in this study (ADA155) is highlighted in red. Circles at branch tips are coloured according to the country of origin. Ultrafast bootstrap support values are shown only for major lineages and external branches with values greater than 80%. Scale bars represent the number of nucleotide substitutions per site. Trees are midpoint-rooted for visualization purposes.

To investigate the genetic diversity of the ADA155 TOSV genome, we conducted ML phylogenetic analyses using publicly available sequences representing the three established TOSV genotypes (A, B and C), generating trees for each genome segment independently (Fig. 4). In all three segments (S, M and L), ADA155 consistently clustered within genotype B. It showed the closest relationship to the Fue-Sp45 strain (GenBank accessions LN848251.1, LN848246.1 and LN848240.1), previously detected in the Madrid region during the 2012–2013 leishmaniasis outbreak [20], with nucleotide homology of 98.5%, 97.5% and 97.5%, respectively.

## Discussion

In the present study, we used vmNGS to identify viral pathogens in the CSF of adults with suspected viral CNS infections who remained undiagnosed after extensive routine diagnostic testing, which primarily included the Meningitis/Encephalitis FilmArray^®^ Panel and standard bacterial and fungal cultures. Viral loads in CSF from patients with CNS infections are typically low [5]. To address this, we used a probe-based hybrid-capture enrichment panel targeting over 3,000 viral genomes from diverse DNA and RNA viral families. This enrichment strategy, combined with RNA-based metagenomic sequencing, has been shown to improve sensitivity for detecting low-abundance viral pathogens in CSF by targeting viral transcripts produced during active replication [8,9, 21]. In our study, this approach enabled the identification of both RNA (HPgV and TOSV) and DNA (HSV-1 and VZV) viruses in 6 of the 40 idiopathic samples. All cases were subsequently confirmed by virus-specific PCR, providing independent validation of the metagenomic results.

HPgV RNA was detected in the CSF of three patients presenting with neurological syndromes, including meningitis, meningoencephalitis and multiple neuritis. HPgV (formerly known as GB virus C or hepatitis G virus) is a positive-sense RNA virus of the *Pegivirus* genus within the *Flaviviridae* family and is commonly transmitted via blood, sexual contact or vertical transmission [22]. It is highly prevalent, with 2–6% of healthy blood donors in developed countries, ~3% in Spain [22,23], and can establish persistent infections in humans, which led to its renaming as ‘human pegivirus’ (for ‘persistent G’ virus). To date, HPgV infection has not been clearly associated with any specific clinical disease. Nevertheless, its occasional detection in CNS-related samples has raised questions about the significance of HPgV RNA in this compartment. Several studies have reported HPgV RNA in CSF of patients with encephalitis, aseptic meningitis, encephalomyelitis, leukoencephalitis, myelitis and other neurological syndromes [24,32]. In addition, experimental data suggest that HPgV can infect neural cell types *in vitro*, including astrocytes [24]. Taken together, these observations indicate that HPgV may occasionally be detected within the CNS compartment, but they do not establish a causal role in neurological disease.

In our study, HPgV RNA was detected in 3 of 40 (7.5%) idiopathic CSF samples. Detection frequencies reported in other metagenomic studies of unexplained CNS disease vary, with HPgV detected in 4 and 3% of CSF samples from comparable cohorts in Brazil and Poland, respectively, whereas a Swiss study with a similar diagnostic approach reported HPgV detection in 8% of cases [29,31]. However, given differences in cohort selection, immune status, sample processing, sequencing methods and bioinformatic criteria across studies, these comparisons should be regarded as descriptive only and not as evidence of increased HPgV detection in our cohort.

Two of the three HPgV-positive patients in our cohort were transplant recipients: one haematopoietic stem cell transplant and one liver transplant recipient. This observation is of interest because HPgV has been reported in the blood virome of immunocompromised individuals, including transplant recipients, and long-term persistence has been described in this population [33,39]. In both transplant recipients, neurological symptoms occurred more than 2 years after transplantation. This timing should not be interpreted as evidence of a causal relationship, but it is relevant given the reported long-term persistence of HPgV in immunocompromised individuals. The presence of HPgV in CSF may therefore reflect several non-mutually exclusive possibilities, including incidental detection during persistent systemic infection, passage of free virus or infected cells across an inflamed blood–brain barrier, blood-derived virus or true but unproven CNS infection. In our cases, HPgV RNA was detected in patients with inflammatory CSF findings, including pleocytosis, supporting the presence of CNS inflammation but not demonstrating that HPgV was the cause. Accordingly, our data cannot determine whether immunosuppression increased the likelihood of HPgV detection in CSF or whether HPgV contributed to the neurological presentation.

In Spain, data on circulating HPgV genotypes remain scarce and restricted to blood donor cohorts [23]. HPgV genotypes show broad geographical structuring, with genotype 2 predominating in Europe and genotype 3 more commonly reported in Asia and South America [40]. In our study, phylogenetic analysis placed ADA18 and ADA63 within genotype 2, consistent with the predominance of this genotype in Europe and with previous data from Spain [23]. The partial ADA46 sequence clustered with genotype 3 reference strains; however, because genome recovery was limited, this assignment should be interpreted cautiously. Expanded genomic surveillance across diverse clinical presentations will be needed to clarify the molecular epidemiology of HPgV in Spain.

A noteworthy finding of our study was the detection of TOSV in a patient residing in the Madrid region (central Spain). The sample was collected in June, coinciding with the typical seasonal peak of TOSV infections. TOSV is an arbovirus transmitted by sandflies that is a leading cause of viral meningitis and meningoencephalitis in Mediterranean regions [41]. In Spain, most reported cases occur in coastal regions such as Catalonia, Murcia, Andalusia and the Balearic Islands, with seroprevalence ranging from 5% to 26% depending on the region [41]. Previous serological studies have also reported evidence of TOSV exposure in Madrid, although estimates differ depending on the population and assay used [42,43]. Because this study included only one TOSV-positive case among a selected group of patients with unresolved CNS disease, our data cannot estimate the prevalence of TOSV infection or determine the extent of TOSV circulation in central Spain. The ADA155 sequence clustered within genotype B and was most closely related to a strain previously detected in Madrid during a 2012–2013 leishmaniasis outbreak [20], rather than to more recent genomes identified in southern Spain during 2015–2019 [4]. The absence of reported recent travel and the phylogenetic relatedness to a previous Madrid strain are compatible with local acquisition. Nevertheless, the detection of TOSV RNA in CSF from a patient sampled during the vector season supports considering TOSV in selected cases of unexplained aseptic meningitis in central Spain, including areas beyond the Mediterranean coastal regions where most Spanish cases have traditionally been reported. Although no specific antiviral therapy is currently available, laboratory confirmation remains relevant for improving aetiological diagnosis, supporting rational clinical management and generating genomic data that may inform future molecular, genomic and entomological surveillance studies.

Another finding of this study was the detection of HSV-1 and VZV in patients with encephalitis and myelitis, respectively, consistent with the well-established fact that HSV-1 is the leading cause of viral encephalitis and that VZV reactivation can sometimes cause myelitis [1]. In our cohort, magnetic resonance imaging findings in the HSV-1 case and cutaneous herpes zoster lesions in the VZV case support a likely association between each virus and its clinical manifestation. However, both viruses were missed by the FilmArray^®^ Meningitis/Encephalitis Panel. The false-negative result for the HSV-1–positive patient was unexpected given the low Ct value (23.7) and the fact that we were able to obtain an almost complete genome, suggesting a high viral load. Nevertheless, false-negative results for both HSV-1 and VZV have been reported previously [9,44, 45], and a recent meta-analysis showed that, despite good overall performance, the FilmArray^®^ panel has suboptimal sensitivity for HSV-1 [46]. While the panel is generally highly sensitive for VZV [46], its failure to detect the virus in this CSF sample is most likely due to the typically low viral load [44], which also likely explains why a complete VZV genome could not be obtained by sequencing. Overall, our findings support the notion that a negative FilmArray^®^ result does not exclude HSV-1 or VZV infection, particularly when clinical suspicion is high. In such situations, additional diagnostic approaches, including conventional PCR, repeat CSF sampling or vmNGS when available, should be considered to ensure accurate diagnosis and guide ongoing antiviral therapy [45,46].

This study has several limitations. First, the small sample size and retrospective design limit the conclusions that can be drawn. The findings should therefore not be interpreted as prevalence estimates or as evidence of causal associations between the detected viruses and neurological disease, but rather as descriptive, sequence-level observations requiring confirmation in larger prospective cohorts. In addition, CSF samples were not collected or stored under conditions optimized for vmNGS; long-term storage at −20 °C may have impaired viral RNA stability and reduced sensitivity. Another limitation of our study is the inherent difficulty of using NGS to determine the clinical relevance of detected viral sequences, particularly for viruses such as HPgV. Detection in the CNS alone does not imply causality, and establishing such an association was not the aim of this study. Future studies, including paired blood and CSF samples, quantitative and longitudinal viral-load assessment, intrathecal immune-response analysis and matched control CSF samples processed with the same workflow, will be required to distinguish incidental viral detection from clinically relevant CNS involvement.

In conclusion, our study demonstrates the added diagnostic value of probe-based hybrid-capture vmNGS for detecting viral genomes in CSF samples from adults with CNS infections unresolved by routine testing. The identification of both unexpected viral detections, such as HPgV and TOSV, and missed treatable infections, including HSV-1 and VZV, supports the use of targeted complementary molecular approaches in selected clinical scenarios. In addition, the recovery and phylogenetic characterization of viral sequences from CSF provide genomic data that may contribute to future studies on the molecular epidemiology of viruses detected in neurological disease in Spain.

## Supplementary material

10.1099/jgv.0.002302 Supplementary Material 1.

## Abbreviations

CMV: cytomegalovirus
CNS: central nervous system
CSF: cerebrospinal fluid
EBV: Epstein-Barr virus
EV: enterovirus
GTR: General Time Reversible
HHV-6: human herpesvirus 6
HHV-7: human herpesvirus 7
HPgV: human pegivirus
HSV-1: herpes simplex virus type 1
HSV-2: herpes simplex virus 2
HUPH: Hospital Universitario Puerta de Hierro
IQR: interquartile range
ML: maximum likelihood
r.p.m.: reads per million
TOSV: Toscana virus
vmNGS: viral metagenomic next-generation sequencing
VZV: varicella-zoster virus

## References

[1] John CC, Carabin H, Montano SM, Bangirana P, Zunt JR (2015). Global research priorities for infections that affect the nervous system. Nature.

[2] Shukla B, Aguilera EA, Salazar L, Wootton SH, Kaewpoowat Q (2017). Aseptic meningitis in adults and children: diagnostic and management challenges. J Clin Virol.

[3] McGill F, Griffiths MJ, Bonnett LJ, Geretti AM, Michael BD (2018). Incidence, aetiology, and sequelae of viral meningitis in UK adults: a multicentre prospective observational cohort study. Lancet Infect Dis.

[4] Gámbaro F, Pérez AB, Prot M, Agüera E, Baidaliuk A (2023). Untargeted metagenomic sequencing identifies Toscana virus in patients with idiopathic meningitis, Southern Spain, 2015 to 2019. Euro Surveill.

[5] Ramachandran PS, Wilson MR (2020). Metagenomics for neurological infections - expanding our imagination. Nat Rev Neurol.

[6] Piantadosi A, Mukerji SS, Ye S, Leone MJ, Freimark LM (2021). Enhanced virus detection and metagenomic sequencing in patients with meningitis and encephalitis. mBio.

[7] Saha S, Ramesh A, Kalantar K, Malaker R, Hasanuzzaman M (2019). Unbiased metagenomic sequencing for pediatric meningitis in bangladesh reveals neuroinvasive chikungunya virus outbreak and other unrealized pathogens. mBio.

[8] Castellot A, Camacho J, Fernández-García MD, Tarragó D (2023). Shotgun metagenomics to investigate unknown viral etiologies of pediatric meningoencephalitis. PLoS One.

[9] Launes C, Camacho J, Pons-Espinal M, López-Labrador FX, Esteva C (2024). Hybrid capture shotgun sequencing detected unexpected viruses in the cerebrospinal fluid of children with acute meningitis and encephalitis. Eur J Clin Microbiol Infect Dis.

[10] Recio V, González I, Tarragó D (2023). Cytomegalovirus drug resistance mutations in transplant recipients with suspected resistance. Virol J.

[11] Fernandez-Garcia MD, Garcia-Ibañez N, Camacho J, Gutierrez A, Sánchez García L (2024). Enhanced echovirus 11 genomic surveillance in neonatal infections in Spain following a European alert reveals new recombinant forms linked to severe cases, 2019 to 2023. Euro Surveill.

[12] Kalantar KL, Carvalho T, de Bourcy CFA, Dimitrov B, Dingle G (2021). IDseq – An open source cloud-based pipeline and analysis service for metagenomic pathogen detection and monitoring. Gigascience.

[13] Mourik K, Sidorov I, Carbo EC, van der Meer D, Boot A (2024). Comparison of the performance of two targeted metagenomic virus capture probe-based methods using reference control materials and clinical samples. J Clin Microbiol.

[14] López-Labrador FX, Brown JR, Fischer N, Harvala H, Van Boheemen S (2021). Recommendations for the introduction of metagenomic high-throughput sequencing in clinical virology, part I: wet lab procedure. J Clin Virol.

[15] RStudio Team (2025). RStudio: Integrated Development Environment for R. Posit Software, PBC, Boston, MA. https://posit.co.

[16] Minh BQ, Schmidt HA, Chernomor O, Schrempf D, Woodhams MD (2020). IQ-TREE 2: new models and efficient methods for phylogenetic inference in the genomic era. Mol Biol Evol.

[17] Kalyaanamoorthy S, Minh BQ, Wong TKF, von Haeseler A, Jermiin LS (2017). ModelFinder: fast model selection for accurate phylogenetic estimates. Nat Methods.

[18] Hoang DT, Chernomor O, von Haeseler A, Minh BQ, Vinh LS (2018). UFBoot2: improving the ultrafast bootstrap approximation. Mol Biol Evol.

[19] Sánchez-Seco M-P, Echevarría J-M, Hernández L, Estévez D, Navarro-Marí J-M (2003). Detection and identification of Toscana and other phleboviruses by RT-nested-PCR assays with degenerated primers. J Med Virol.

[20] Remoli ME, Jiménez M, Fortuna C, Benedetti E, Marchi A (2016). Phleboviruses detection in *Phlebotomus perniciosus* from a human leishmaniasis focus in South-West Madrid region, Spain. Parasit Vectors.

[21] Carbo EC, Buddingh EP, Karelioti E, Sidorov IA, Feltkamp MCW (2020). Improved diagnosis of viral encephalitis in adult and pediatric hematological patients using viral metagenomics. J Clin Virol.

[22] Stapleton JT (2022). Human pegivirus type 1: a common human virus that is beneficial in immune-mediated disease?. Front Immunol.

[23] Cebriá-Mendoza M, Bracho MA, Arbona C, Larrea L, Díaz W (2021). Exploring the diversity of the human blood virome. Viruses.

[24] Doan MAL, Roczkowsky A, Smith M, Blevins G, van Landeghem FKH (2021). Infection of glia by human pegivirus suppresses peroxisomal and antiviral signaling pathways. J Virol.

[25] Tuddenham R, Eden J-S, Gilbey T, Dwyer DE, Jennings Z (2020). Human pegivirus in brain tissue of a patient with encephalitis. Diagn Microbiol Infect Dis.

[26] Balcom EF, Doan MAL, Branton WG, Jovel J, Blevins G (2018). Human pegivirus-1 associated leukoencephalitis: clinical and molecular features. Ann Neurol.

[27] Valyraki N, Maillart E, Pourcher V, Shor N, Tran S (2023). Human pegivirus identified in severe myelitis and optic neuritis in immunocompromised patients: a pathogenic role for a forgotten virus?. Rev Neurol.

[28] Fridholm H, Østergaard Sørensen L, Rosenstierne MW, Nielsen H, Sellebjerg F (2016). Human pegivirus detected in a patient with severe encephalitis using a metagenomic pan-virus array. J Clin Virol.

[29] Carmona R de CC, Cilli A, da Costa AC, Reis FC, Leal É (2024). Pegivirus detection in cerebrospinal fluid from patients with central nervous system infections of unknown etiology in brazil by viral metagenomics. Microorganisms.

[30] Bukowska-Ośko I, Perlejewski K, Pawełczyk A, Rydzanicz M, Pollak A (2018). Human pegivirus in patients with encephalitis of unclear etiology, Poland. Emerg Infect Dis.

[31] Pichler I, Krischer E, Obenhuber T, Hirsch B, Reinhold I (2026). Viral metagenomic sequencing reveals rare pathogens and improves diagnostic accuracy in neuroinflammatory disorders. Diagn Microbiol Infect Dis.

[32] Scheibe F, Melchert J, Radbruch H, Siebert E, Best TD (2025). Pegivirus-associated encephalomyelitis in immunosuppressed patients. N Engl J Med.

[33] Li Z, Li Y, Liang Y, Hu L, Chen S (2019). Prevalence and risk factors of human pegivirus type 1 infection in hematopoietic stem cell transplantation patients. Int J Infect Dis.

[34] Ludowyke N, Phumiphanjarphak W, Apiwattanakul N, Manopwisedjaroen S, Pakakasama S (2022). Target enrichment metagenomics reveals human pegivirus-1 in pediatric hematopoietic stem cell transplantation recipients. Viruses.

[35] Zanella M-C, Vu D-L, Hosszu-Fellous K, Neofytos D, Van Delden C (2023). Longitudinal detection of twenty DNA and RNA viruses in allogeneic hematopoietic stem cell transplant recipients plasma. Viruses.

[36] Izumi T, Sakata K, Okuzaki D, Inokuchi S, Tamura T (2019). Characterization of human pegivirus infection in liver transplantation recipients. J Med Virol.

[37] Zanella MC, Cordey S, Laubscher F, Docquier M, Vieille G (2021). Unmasking viral sequences by metagenomic next-generation sequencing in adult human blood samples during steroid-refractory/dependent graft-versus-host disease. Microbiome.

[38] Zanella MC, Cordey S, Kaiser L (2020). Beyond cytomegalovirus and epstein-barr virus: a review of viruses composing the blood virome of solid organ transplant and hematopoietic stem cell transplant recipients. Clin Microbiol Rev.

[39] Vu D-L, Cordey S, Simonetta F, Brito F, Docquier M (2019). Human pegivirus persistence in human blood virome after allogeneic haematopoietic stem-cell transplantation. Clin Microbiol Infect.

[40] Yu Y, Wan Z, Wang JH, Yang X, Zhang C (2022). Review of human pegivirus: prevalence, transmission, pathogenesis, and clinical implication. Virulence.

[41] García San Miguel L, Sierra MJ, Vazquez A, Fernandez-Martínez B, Molina R (2021). Phlebovirus-associated diseases transmitted by phlebotominae in Spain: are we at risk?. Enfermedades Infecc y Microbiol Clin.

[42] Echevarría J-M, de Ory F, Guisasola M-E, Sánchez-Seco M-P, Tenorio A (2003). Acute meningitis due to Toscana virus infection among patients from both the Spanish Mediterranean region and the region of Madrid. J Clin Virol.

[43] Martinez JG, García SG, Walter S, Gil-Prieto R, Lacomba DL (2022). Seroprevalence against Toscana virus in Spain: the case of the autonomous community of Madrid. J Vector Borne Dis.

[44] Lindström J, Elfving K, Lindh M, Westin J, Studahl M (2022). Assessment of the FilmArray ME panel in 4199 consecutively tested cerebrospinal fluid samples. Clin Microbiol Infect.

[45] Schnuriger A, Vimont S, Godmer A, Gozlan J, Gallah S (2022). Differential performance of the FilmArray Meningitis/Encephalitis assay to detect bacterial and viral pathogens in both pediatric and adult populations. Microbiol Spectr.

[46] Trujillo-Gómez J, Tsokani S, Arango-Ferreira C, Atehortúa-Muñoz S, Jimenez-Villegas MJ (2022). Biofire FilmArray Meningitis/Encephalitis panel for the aetiological diagnosis of central nervous system infections: a systematic review and diagnostic test accuracy meta-analysis. eClinicalMedicine.

