# Supplementary material for: Human pegivirus, Toscana virus and herpesviruses identified in cerebrospinal fluid from adults with unexplained neurologic disease, Spain, 2022–2023
Source: J Gen Virol. 2026 Jul 17;107(7):002302. doi: 10.1099/jgv.0.002302 (PMC13379243; doi:10.1099/jgv.0.002302)

Supplementary Material

Human Pegivirus, Toscana Virus and Herpesviruses Identified in Cerebrospinal Fluid from Adults with Unexplained Neurologic Disease, Spain, 2022-2023

Supplementary Table 1. Summary of clinical and demographic data of patients with suspected neurological infections enrolled for viral mNGS analysis of CSF samples after negative results using clinical routine diagnostic methods. Also shown are the mNGS sequencing results (n=40). [a]Hematopoietic stem cell transplant recipient; [b]Liver transplant recipient; Age-group (years); Abbreviations: M: male; F: female; CSF: cerebrospinal fluid; FA-ME: FilmArray® Meningitis/Encephalitis Panel; BCU: CSF bacterial culture; FCU: CSF fungal culture; EV: enterovirus; HSV: Herpes Simplex Virus; HHV: Human Herpesvirus; WNV: West Nile Virus; VZV: Varicella Zoster Virus; CMV: Cytomegalovirus; EBV: Epstein-Barr virus; Hospital Universitario Puerta de Hierro (HUPH) in Madrid region; Hospital Universitario Reina Sofia (HURS) in Andalusia.

Hospital	Lab ID	Age group	Sex	Clinical suspicion	Routine Clinical Laboratory tests	Total Number Reads	% host reads	Virus Hit*
HURS	ADA36	20-29	M	Meningoencephalitis	BCU; FA-ME	5925004	22.84	
HURS	ADA37	40-49	F	Encephalitis	BCU; FCU; FA-ME; Toxoplasma PCR	1664188	82.34	
HURS	ADA38	60-69	F	Encephalitis	BCU; FCU; FA-ME; PCR and serology for WNV and Toscana virus in CSF, serum, and urine	37912482	78.28	
HURS	ADA39	60-69	M	Meningoencephalitis		6401372	77.00	
HURS	ADA41	60-69	M	Encephalitis	BCU; FCU; FA-ME	10651330	14.56	HSV-1
HURS	ADA42	30-39	M	Encephalitis	BCU; FA-ME; PCR for WNV in CSF	29384260	93.02	
HURS	ADA46[a]	50-59	M	Meningoencephalitis	BCU; FCU; FA-ME; Cryptococcal antigen	2438928	94.91	Pegivirus
HURS	ADA48	70-79	M	Meningitis	BCU; FCU; FA-ME	3819912	98.36	
HURS	ADA49	20-29	M	Myelitis	BCU; FCU; FA-ME; IgG serology for HSV-1, HSV-2, and VZV in CSF	3027508	98.34	
HURS	ADA56	30-39	M	Myelitis	BCU; FCU; HSV, VZV, EV PCRs	19135386	84.40	
HURS	ADA58	40-49	M	Meningitis	HSV, VZV PCRs	1832814	98.22	
HURS	ADA60	60-69	M	Meningoencephalitis	BCU; FCU; FA-ME; PCR and serology for WNV and Toscana virus in CSF, serum, and urine	3260092	96.47	
HURS	ADA63	20-29	F	Meningitis	BCU; FCU; FA-ME	1076670	96.83	Pegivirus
HURS	ADA66	80-89	F	Meningoencephalitis	BCU; FCU; FA-ME	2485326	98.55	
HURS	ADA67	70-79	M	Meningoencephalitis	BCU; FCU; FA-ME	2194940	84.16	
HURS	ADA68	50-59	F	Myelitis	BCU; FCU; FA-ME	3486140	97.95	
HURS	ADA70	60-69	F	Encephalitis	BCU; FCU; FA-ME	3146326	96.68	
HURS	ADA78	60-69	M	Meningitis	BCU; FCU; FA-ME	25555316	96.59	
HURS	ADA79	30-39	M	Meningitis	BCU; FCU; FA-ME	23882792	95.09	
HURS	ADA80	30-39	M	Meningitis	BCU; FCU; FA-ME; PCR and serology for WNV and Toscana virus in CSF, serum, and urine	993380	97.04	
HURS	ADA112	40-49	M	Myelitis	BCU; FCU; FA-ME	17679688	78.48	VZV
HURS	ADA114	70-79	M	Meningitis	BCU; FCU; FA-ME	27967526	69.15	
HUPH	ADA1	60-69	F	Inflammatory brain lesions	FA-ME	186208	31.10	
HUPH	ADA2	40-49	M	Encephalitis	BCU; EBV, CMV, HSV, VZV, EV, HHV-6-7-8 PCRs	113516	27.53	
HUPH	ADA3	70-79	M	Radiculoneuritis	BCU; EBV, HSV, VZV, EV, HHV-6 PCRs	43201942	57.86	
HUPH	ADA4	70-79	F	Pseudotumoral syndrome	BCU; FCU; EBV, CMV, HSV, VZV, EV, HHV-6-7-8, JCV PCRs	10339390	31.06	
HUPH	ADA5	60-69	M	Encephalopathy	BCU; EBV, CMV, HSV, VZV, EV, HHV-6 PCRs	14574396	47.91	
HUPH	ADA6	50-59	M	Encephalopathy	BCU; FA-ME	61713670	30.74	
HUPH	ADA7	70-79	F	Meningitis	HSV, VZV, EV PCRs	549734	36.31	
HUPH	ADA9	50-59	M	Acute symptomatic seizure	BCU; EBV, CMV, HSV, VZV, HHV-6 PCRs	50390014	20.50	
HUPH	ADA18[b]	60-69	M	Multiple neuritis	BCU; FA-ME	602428	54.22	Pegivirus
HUPH	ADA83	40-49	M	Meningitis	BCU; FA-ME; Cryptococcal antigen	28504732	97.46	
HUPH	ADA84	50-59	F	Encephalitis	BCU; EBV, CMV, HSV, VZV, EV, HHV-6-7-8 PCRs	6803040	69.32	
HUPH	ADA85	20-29	M	Encephalitis	BCU; EBV, CMV, HSV, VZV, EV, HHV-6-7-8 PCRs	29669234	81.11	
HUPH	ADA91	70-79	M	Possible inflammatory cerebellar lesion	EBV, CMV, HHV-6 PCRs	976288	35.27	
HUPH	ADA94	40-49	M	Myelitis	BCU; EBV, VHS, VZV, EV, HHV-6, CMV PCRs	387942	19.81	
HUPH	ADA99	70-79	M	Polyneuropathy	EBV; HSV; VZV; HHV-6-7-8, CMV PCRs	713496	5.83	
HUPH	ADA100	70-79	M	Myelitis	BCU; EBV, HSV, VZV, CMV, EV PCRs	585478	12.62	
HUPH	ADA103	60-69	F	Encephalitis	EBV, CMV, HSV, VZV, EV, HHV-6-7-8 PCRs	217854	18.14	
HUPH	ADA155	60-69	F	Meningitis	BCU; FA-ME; EBV, CMV, HSV, VZV, EV, HHV-6-7-8 PCRs	3422946	39.68	Toscana Virus

Supplementary Figure 1. Amplification of A) human pegivirus (HPgV) RNA B) Varicella-Zoster virus (VZV) DNA C) Toscana virus (TOSV) RNA D) Herpes Simplex Virus 1 (HSV-1) DNA from CSF clinical samples in which these viruses were detected by mNGS. The expected amplicon sizes were approximately 322 bp for HPgV, 116 bp for VZV and 244 for TOSV. Electrophoresis of the PCR products on a 2% agarose gel demonstrated specific bands in samples ADA18, ADA63, ADA46, ADA112, and ADA155. Amplicons were Sanger-sequenced and confirmed to correspond to HPgV, VZV, and TOSV. For the TOSV panel (C), a sample positive for a related phlebovirus was included as a negative control, explaining the presence of a band. Ladder: molecular weight marker.

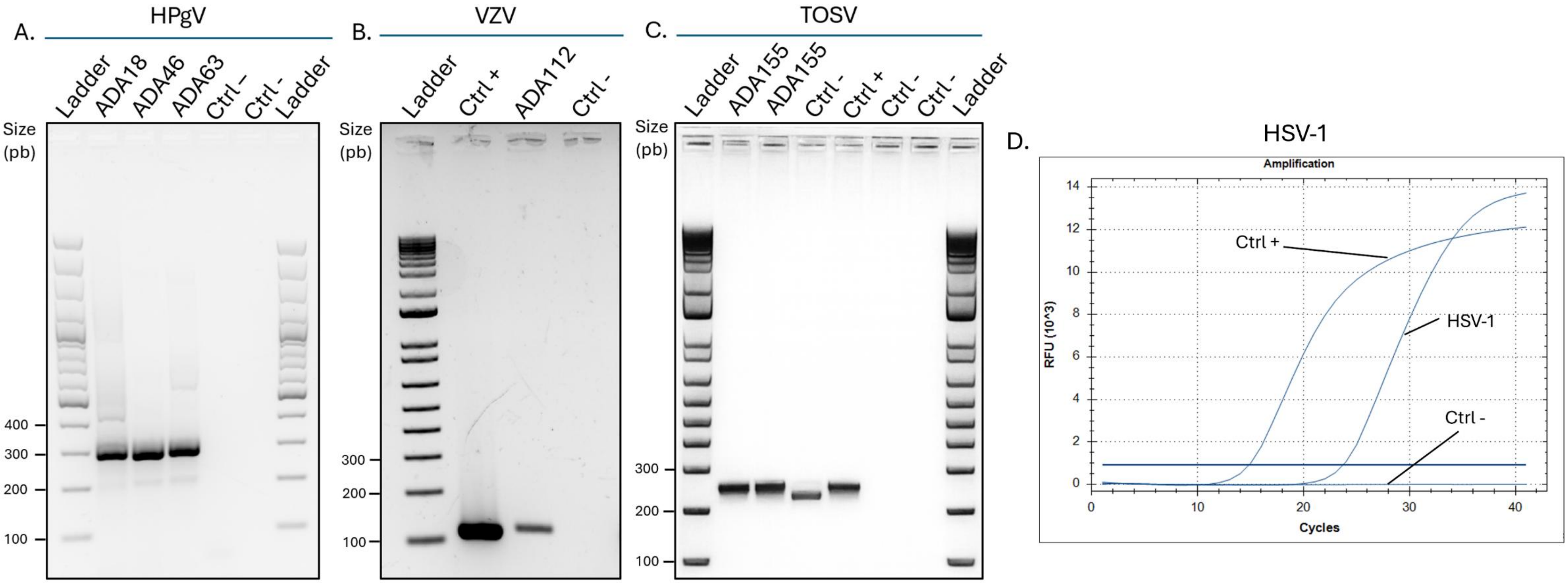

A.
HPgV
Ladder
ADA18
ADA46
ADA63
Ctrl –
Ctrl -
Ladder
Size (pb)
400
300
200
100
B.
VZV
Ladder
Ctrl +
ADA112
Ctrl -
Size (pb)
300
200
100
C.
TOSV
Ladder
ADA155
ADA155
Ctrl -
Ctrl +
Ctrl -
Ctrl -
Ladder
Size (pb)
300
200
100
D.
HSV-1
Amplification
RFU (10^3)
Cycles
Ctrl +
HSV-1
Ctrl -
0
2
4
6
8
10
12
14
0
10
20
30
40

Supplementary Figure 2. Non-collapsed Maximum likelihood phylogenetic tree based on the complete genome of the human pegivirus (HPgV). The tree was constructed using HPgV sequences from GenBank, including virus strains identified by vmNGS in this study. Bootstrapping (1,000 replicates) was used to assess node support, and the General Time Reversible model was applied as the substitution model. Genotypes at the nodes are shown as circles, and bootstrap values are shown for each genotype cluster. ADA63, ADA46 and ADA18 are shown as red stars.

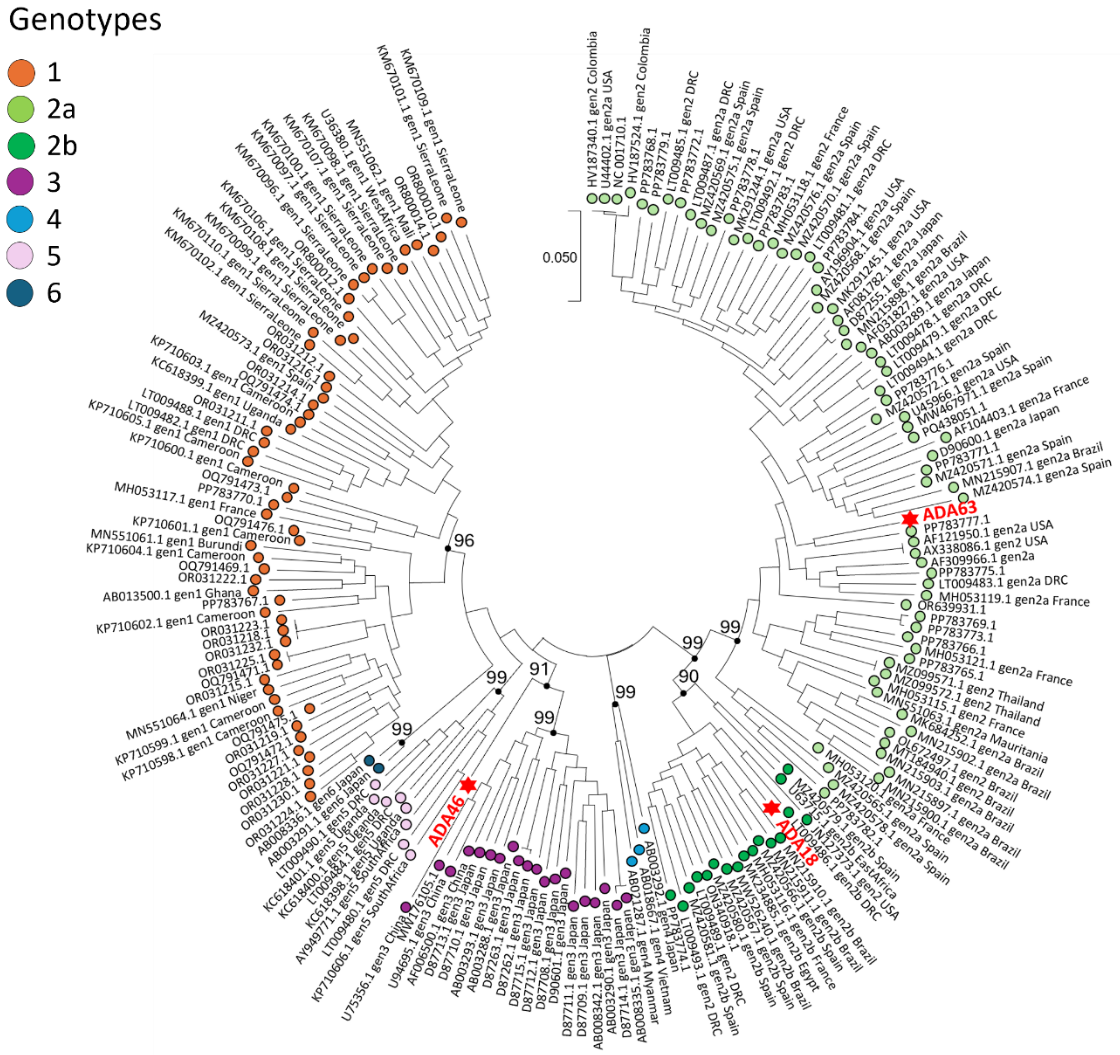

Supplement: Supplementary Material 1. [file jgv-107-02302-s001.pdf]
